# Think globally, measure locally: The MIREN standardized protocol for monitoring plant species distributions along elevation gradients

**DOI:** 10.1002/ece3.8590

**Published:** 2022-02-14

**Authors:** Sylvia Haider, Jonas J. Lembrechts, Keith McDougall, Aníbal Pauchard, Jake M. Alexander, Agustina Barros, Lohengrin A. Cavieres, Irfan Rashid, Lisa J. Rew, Alla Aleksanyan, José R. Arévalo, Valeria Aschero, Chelsea Chisholm, V. Ralph Clark, Jan Clavel, Curtis Daehler, Pervaiz A. Dar, Hansjörg Dietz, Romina D. Dimarco, Peter Edwards, Franz Essl, Eduardo Fuentes‐Lillo, Antoine Guisan, Onalenna Gwate, Anna L. Hargreaves, Gabi Jakobs, Alejandra Jiménez, Paul Kardol, Christoph Kueffer, Christian Larson, Jonathan Lenoir, Bernd Lenzner, Miguel A. Padrón Mederos, Maritza Mihoc, Ann Milbau, John W. Morgan, Jana Müllerová, Bridgett J. Naylor, Ivan Nijs, Martin A. Nuñez, Rüdiger Otto, Niels Preuk, Amanda Ratier Backes, Zafar A. Reshi, Sabine B. Rumpf, Verónica Sandoya, Mellesa Schroder, Karina L. Speziale, Davnah Urbach, Graciela Valencia, Vigdis Vandvik, Michaela Vitková, Tom Vorstenbosch, Tom W. N. Walker, Neville Walsh, Genevieve Wright, Shengwei Zong, Tim Seipel

**Affiliations:** ^1^ Institute of Biology/Geobotany and Botanical Garden Martin Luther University Halle‐Wittenberg Halle Germany; ^2^ 530625 German Centre for Integrative Biodiversity Research (iDiv) Halle‐Jena‐Leipzig Leipzig Germany; ^3^ 26660 Research group Plants and Ecosystems (PLECO) University of Antwerp Wilrijk Belgium; ^4^ Department of Planning, Industry and Environment Queanbeyan New South Wales Australia; ^5^ 28056 Laboratorio de Invasiones Biologicas (LIB) Facultad de Ciencias Forestales Universidad de Concepción Concepción Chile; ^6^ Institute of Ecology and Biodiversity (IEB) Santiago Chile; ^7^ 27219 Institute of Integrative Biology ETH Zürich Zürich Switzerland; ^8^ Instituto Argentino de Nivología y Glaciología y Ciencias Ambientales (IANIGLA) Centro Científico Tecnológico (CCT) CONICET Mendoza Mendoza Argentina; ^9^ 28056 Departamento de Botánica Facultad de Ciencias Naturales y Oceanográficas Universidad de Concepción Concepción Chile; ^10^ 29079 Department of Botany University of Kashmir Srinagar India; ^11^ Department of Land Resource and Environmental Sciences Montana State University Bozeman Montana USA; ^12^ Department of Geobotany and Plant Ecophysiology Institute of Botany aft. A.L. Takhtajyan NAS RA Yerevan Armenia; ^13^ 230738 Chair of Biology and Biotechnologies Armenian National Agrarian University Yerevan Armenia; ^14^ 16749 Department of Botany, Ecology and Plant Physiology University of La Laguna La Laguna Spain; ^15^ Afromontane Research Unit & Department of Geography University of the Free State: Qwaqwa Campus Phuthaditjhaba South Africa; ^16^ School of Life Sciences University of Hawai'i at Manoa Honolulu Hawaii USA; ^17^ Department of Botany Amar Singh College Srinagar India; ^18^ Grupo de Ecología de Poblaciones de Insectos IFAB (INTA‐CONICET) Bariloche Argentina; ^19^ Department of Biology and Biochemistry University of Houston Houston Texas USA; ^20^ Bioinvasions, Global Change, Macroecology Group Department of Botany and Biodiversity Research University of Vienna Vienna Austria; ^21^ School of Education and Social Sciences Adventist University of Chile Chillán Chile; ^22^ 27213 Institute of Earth Surface Dynamics & Department of Ecology and Evolution University of Lausanne Lausanne Switzerland; ^23^ 5620 Department of Biology McGill University Montreal Quebec Canada; ^24^ Department of Forest Ecology and Management Swedish University of Agricultural Sciences Umeå Sweden; ^25^ 26697 Department of Botany and Zoology Centre for Invasion Biology Stellenbosch University Matieland South Africa; ^26^ UR “Ecologie et Dynamique des Systèmes Anthropisés” (EDYSAN UMR 7058 CNRS) Université de Picardie Jules Verne Amiens France; ^27^ Research Institute for Nature and Forest – INBO Brussels Belgium; ^28^ Department of Ecology Environment and Evolution La Trobe University Bundoora Victoria Australia; ^29^ Department of GIS and Remote Sensing Institute of Botany of the Czech Academy of Sciences Průhonice Czech Republic; ^30^ US Forest Service PNW Research Station La Grande Oregon USA; ^31^ Grupo Ecología de Invasiones Instituto de Investigaciones en Biodiversidad y Medio Ambiente CONICET ‐ Universidad Nacional del Comahue Bariloche Argentina; ^32^ 27213 Department of Ecology and Evolution University of Lausanne Lausanne Switzerland; ^33^ 27213 Department of Environmental Sciences University of Basel Basel Switzerland; ^34^ School of Life Sciences and Biotechnology Yachay Tech University Urcuquí Ecuador; ^35^ 16719 CREAF Cerdanyola del Vallès Spain; ^36^ 16719 Unitat d'Ecologia Universitat Autònoma de Barcelona Cerdanyola del Vallès Spain; ^37^ Department of Planning, Industry and Environment Jindabyne New South Wales Australia; ^38^ Laboratorio Ecotono INIBIOMA (CONICET‐UNCOMA) Bariloche Argentina; ^39^ 27210 Global Mountain Biodiversity Assessment Institute of Plant Sciences University of Bern Bern Switzerland; ^40^ Department of Biological Sciences University of Bergen Bergen Norway; ^41^ Department of Invasion Ecology Institute of Botany of the Czech Academy of Sciences Průhonice Czech Republic; ^42^ 4496 Institute of Biology Leiden Leiden University Leiden The Netherlands; ^43^ Institute of Biology University of Neuchâtel Neuchâtel Switzerland; ^44^ 95954 Royal Botanic Gardens Victoria Melbourne Victoria Australia; ^45^ Department of Planning, Industry and Environment NSW Government, Biodiversity and Conservation Queanbeyan New South Wales Australia; ^46^ 47821 Key Laboratory of Geographical Processes and Ecological Security in Changbai Mountains Ministry of Education School of Geographical Sciences Northeast Normal University Changchun China

**Keywords:** climate change, invasive species, long‐term ecological monitoring, MIREN, mountain biodiversity, Mountain Invasion Research Network, range dynamics, range expansions

## Abstract

Climate change and other global change drivers threaten plant diversity in mountains worldwide. A widely documented response to such environmental modifications is for plant species to change their elevational ranges. Range shifts are often idiosyncratic and difficult to generalize, partly due to variation in sampling methods. There is thus a need for a standardized monitoring strategy that can be applied across mountain regions to assess distribution changes and community turnover of native and non‐native plant species over space and time. Here, we present a conceptually intuitive and standardized protocol developed by the Mountain Invasion Research Network (MIREN) to systematically quantify global patterns of native and non‐native species distributions along elevation gradients and shifts arising from interactive effects of climate change and human disturbance. Usually repeated every five years, surveys consist of 20 sample sites located at equal elevation increments along three replicate roads per sampling region. At each site, three plots extend from the side of a mountain road into surrounding natural vegetation. The protocol has been successfully used in 18 regions worldwide from 2007 to present. Analyses of one point in time already generated some salient results, and revealed region‐specific elevational patterns of native plant species richness, but a globally consistent elevational decline in non‐native species richness. Non‐native plants were also more abundant directly adjacent to road edges, suggesting that disturbed roadsides serve as a vector for invasions into mountains. From the upcoming analyses of time series, even more exciting results can be expected, especially about range shifts. Implementing the protocol in more mountain regions globally would help to generate a more complete picture of how global change alters species distributions. This would inform conservation policy in mountain ecosystems, where some conservation policies remain poorly implemented.

## Graphical abstract

We summarize the findings achieved with the standardized sampling protocol developed by the Mountain Invasion Research Network (MIREN) for monitoring the impact of global change on elevational plant species distributions. We intend to promote the use of the protocol to generate global insights into native and non‐native species responses to rapid global change in mountains.

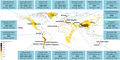

## 1 INTRODUCTION

Mountains are biodiversity hotspots and provide a wealth of ecosystem functions and benefits to people (Körner & Spehn, 2002; Martín‐López et al., 2019; Mengist et al., 2020). At the same time, mountain ecosystems are particularly susceptible to global change. For instance, temperatures are increasing faster at high elevation than at low elevation (Nogués‐Bravo et al., 2007; Pepin et al., 2015). In the alpine zone of the European Alps, temperatures have increased approximately twice as much as the northern hemisphere average over the past 100 years (Gobiet et al., 2014). Importantly, amplified warming has enabled many plant species to move to higher elevation (Lenoir et al., 2008; Pauli et al., 2012; Steinbauer et al., 2018). For instance, between 1971 and 1993, native plant species from the forest understorey in the French mountains shifted their elevational range uphill at an average rate of 38 m per decade (Lenoir et al., 2008). Another prominent example is the observed upward shift of most vascular taxa at Chimborazo in Ecuador since Alexander von Humboldt's visit more than two centuries ago (Morueta‐Holme et al., 2015). An expected consequence of such uphill migrations of more competitive lowland species is that less competitive alpine species might locally become extinct on mountain summits (Alexander et al., 2018; Dullinger et al., 2012; Guisan et al., 2019; Rumpf et al., 2019). Such local extinctions were recently documented for birds (e.g., Freeman et al., 2018).

In addition to temperature increase, human activities in mountain areas have changed markedly over the last decades (e.g., Peters et al., 2019; Wang et al., 2019; for an overview see Payne et al., 2020). Mountain land use has intensified in many places across the globe (Spehn et al., 2006), driven by booming tourism industries (Debarbieux et al., 2014; Pickering & Barros, 2012), overexploitation of natural resources and ever‐increasing demands for agricultural land (e.g., Gillet et al., 2016; Ross et al., 2017). The abandonment of traditional cutting and grazing practices has also occurred in some mountain regions (e.g., MacDonald et al., 2000). Both land use intensification and abandonment can alter plant species distributions and diversity alone (Alexander et al., 2016; Pellissier et al., 2013) and by interacting with climate change (Elsen et al., 2020; Guo et al., 2018).

Further, previously remote areas are becoming increasingly accessible due to construction of new roads and trails, which not only cause a direct disturbance but also act as corridors for plant species movements (Ansong & Pickering, 2013; Lembrechts et al., 2017; Rew et al., 2018). The role of roads as dispersal corridors is amplified due to increased vehicle traffic, often as a result of recreation and tourism (e.g., Müllerová et al., 2011). Roadside habitats also provide ideal spaces for non‐native plants, which generally benefit from reduced competition, increased soil nutrients, more favorable microclimatic and hydrological conditions, and intermediate disturbance (Averett et al., 2016; Müllerová et al., 2011). Thus, both native and non‐native plant species are known to disperse along mountain roads, from low to high elevation and vice versa (Dainese et al., 2017; Guo et al., 2018; Lembrechts et al., 2017). Indeed, many high elevation areas once free of lowland and non‐native species but connected to lowlands by road networks are now harboring lowland and non‐native plant species. Examples for this are the volcanoes of the Hawaiian archipelago (Jakobs et al., 2010), the high Andes (Barros et al., 2020) and the Teide National Park on Tenerife (Dickson et al., 1987). Roadside habitats are also conduits for non‐native plants to spread into natural vegetation once established along roadsides (Alexander et al., 2011; Seipel et al., 2012).

The elevational redistribution of plant species, especially non‐native species (Dainese et al., 2017), has already significantly impacted mountain ecosystems (Guo et al., 2018) and will continue to do so in the future (Petitpierre et al., 2016). For example, non‐native plants can cause biotic homogenization (Haider et al., 2018), reduce the diversity of local native species (Daehler, 2005), and affect important ecosystem functions and services (McDougall, Khuroo, et al., 2011; Pecl et al., 2017). In the mountains of Iceland, non‐native *Lupinus nootkatensis* competes strongly with native plant species and modifies soil properties through nitrogen fixation (Wasowicz, 2016). In the alpine zone of the central Chilean Andes, non‐native *Taraxacum officinale* shares pollinators with several native Asteraceae species (Muñoz & Cavieres, 2019), reducing pollinator‐visitation rates and seed‐set where *T*. *officinale* is at high abundances (Muñoz & Cavieres, 2008). Finally, uphill migration of non‐native trees and shrubs can increase fire risk at high elevation (Cóbar‐Carranza et al., 2014), and transform plant communities through competition (Nuñez et al., 2017; Zong et al., 2016).

While human‐driven vegetation change can happen relatively quickly in mountains, it often only becomes apparent at temporal scales beyond the few years covered by most ecological experiments (Mirtl et al., 2018). Thus, data from long‐term time series in mountains are essential to identify and follow changes in plant communities (Pauli et al., 2012). There are currently two main types of initiatives which monitor high‐elevation vegetation change. At the local or regional scale, some well‐established long‐term monitoring sites follow a holistic approach and document not only floristic changes but also modifications for example of soil, hydrology, or atmospheric conditions. Examples are Niwot Ridge in the Colorado Rocky Mountains (www.nwt.lternet.edu) or the Sierra Nevada Global Change Observatory in Spain (https://obsnev.es/en/). At the global scale, the Global Observation Research Initiative in Alpine Environments (GLORIA, www.gloria.ac.at; Pauli et al., 2015) is a network monitoring floristic change on mountain summits with a standardized approach. What would complement these highly valuable approaches, is a global long‐term monitoring network that covers the full vertical extents of different mountain regions and that allows the detection of species responses to both climate and other human activities.

Here, we present a standardized protocol for monitoring changes in the elevational distribution, abundance, and composition of plant biodiversity in mountains as a result of the interaction between climate and human pressures. Importantly, the protocol focuses on large elevation gradients (>1700 m on average; ranging from c. 700 m to >4000 m), allowing vegetation change to be monitored across a broad range of climates and plant community types. It explicitly contrasts anthropogenically disturbed and (semi‐)natural vegetation within sampling sites, thus increasing detection of rapid community changes and providing greater insight into the drivers of change. The protocol has been developed by the Mountain Invasion Research Network (MIREN, www.mountaininvasions.org) (Kueffer et al., 2014), a network initially founded in 2005 to study patterns and processes of non‐native plant invasions in mountains and recently expanded to more widely understand the effects of global change on mountain plant biodiversity and the distribution of species. The protocol provides a conceptually intuitive yet comprehensive and standardized way to record and monitor native and non‐native species along elevation gradients. The survey has been running in some mountain regions of the world since 2007 (Alexander et al., 2011; Seipel et al., 2012) and continues to be implemented in new regions. In this paper, we summarize the most important findings gained over the time by using this protocol, discuss its strengths and limitations, and outline opportunities and challenges for future work. To achieve broad reach and long‐term maintenance of sites, monitoring protocols must be simple, efficient, and inexpensive. Our intention is to promote the use of the MIREN road survey protocol to monitor biodiversity change in mountains, and to generate global, regional, and local insights into how plant species and communities are responding to rapid global change in mountains.

## 2 MATERIALS AND METHODS

### 2.1 The mountain invasion research network

The Mountain Invasion Research Network (MIREN, www.mountaininvasions.org) was founded in 2005 as a first global effort to apply the known principles from plant invasion ecology in mountainous environments (Kueffer et al., 2008, 2009). From the start, the main goal has been to link detailed observations at the local‐scale from a broad range of mountain regions, to come to global conclusions on common patterns (and divergence from them) regarding mountain plant biodiversity (Kueffer et al., 2014). The core of the network has been the underlying road survey protocol, which allowed flexible application all across the world, yet a standardized baseline of data collection that could be maintained for a long time. While research topics and techniques have diverged throughout the years, the core business of MIREN remains to increase the spatial and temporal extent of the road survey.

### 2.2 Survey design

**FIGURE 1:**
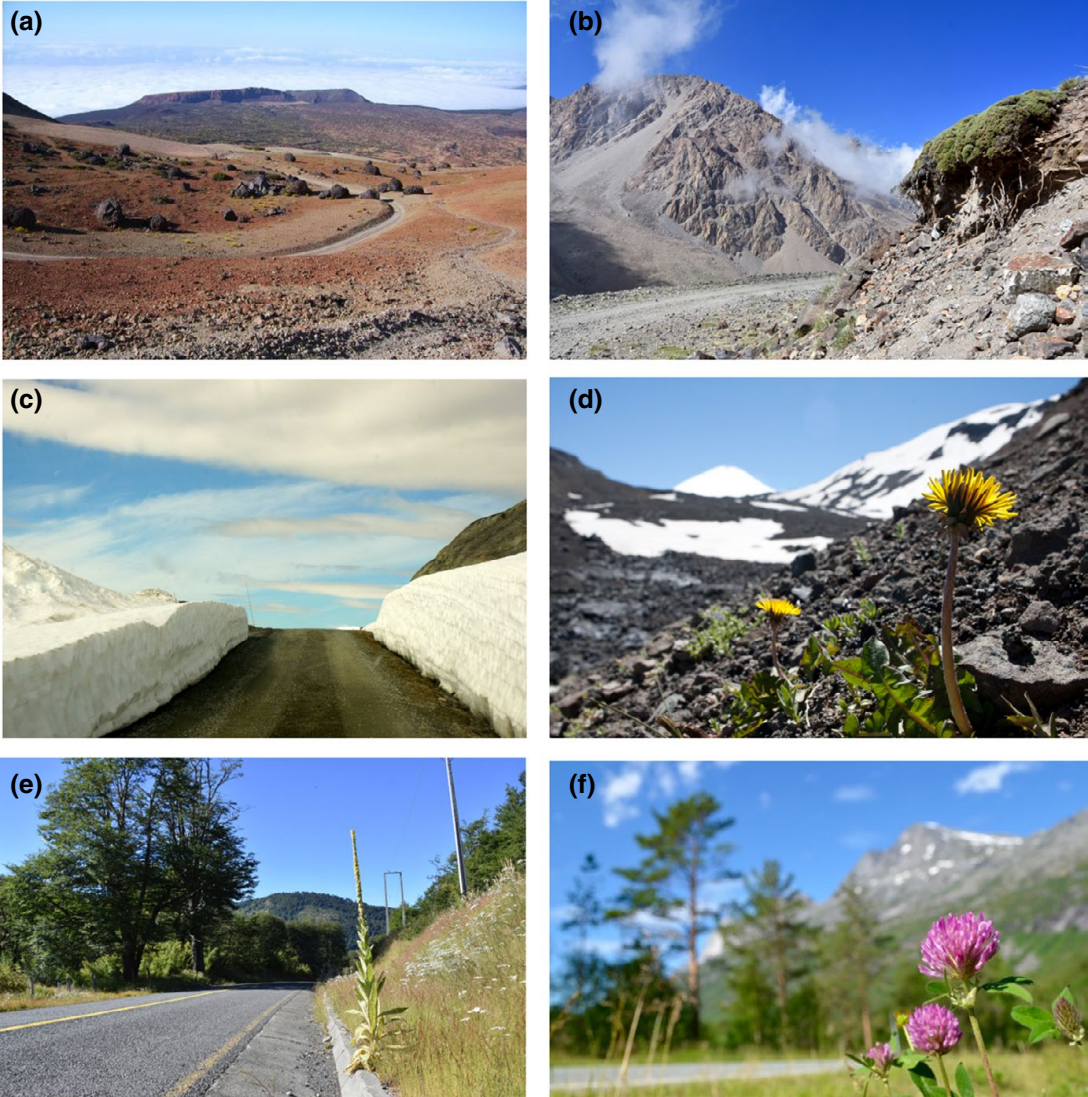
Examples of roads in the landscape (a–c) and key non‐native species (d–f) across a range of MIREN regions. (a) Harsh mountain climates (here the Cañadas del Teide on Tenerife (Canary Islands, Spain) have traditionally been seen as an adequate barrier against non‐native plant invasion; (b) the direct local impact of roadside disturbance on mountain plants is visible on native *Azorella* cushion plants along a road in the dry Andes near Mendoza, Argentina; (c) interactive effects of climate and land use, exemplified by dramatic differences in snow cover on versus beside a mountain road in northern Norway; (d) *Taraxacum officinale*, one of the most widespread non‐native plant species along MIREN mountain roads (Seipel et al., 2012), in a sample plot on a volcanic gravel slope in the Argentine Andes; (e) non‐native *Verbascum thapsus* on a roadside in the highly invaded lowlands of the Andes in central Chile; (f) *Trifolium pratense* in northern Norway, where the species is rapidly moving uphill along mountain roadsides

**FIGURE 2:**
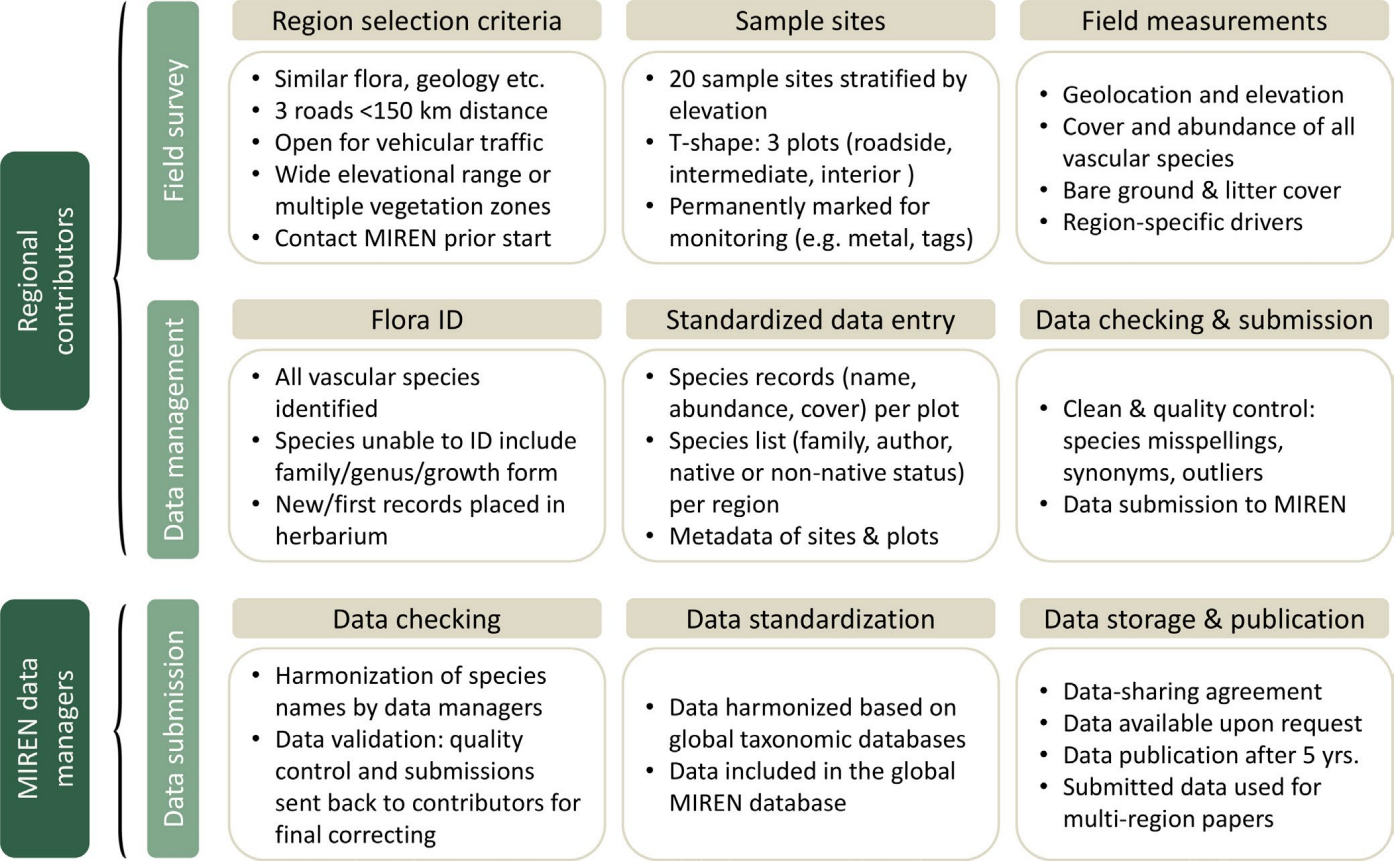
Overview of the workflow from region selection and data collection to inclusion of the data in the global MIREN database

**FIGURE 3:**
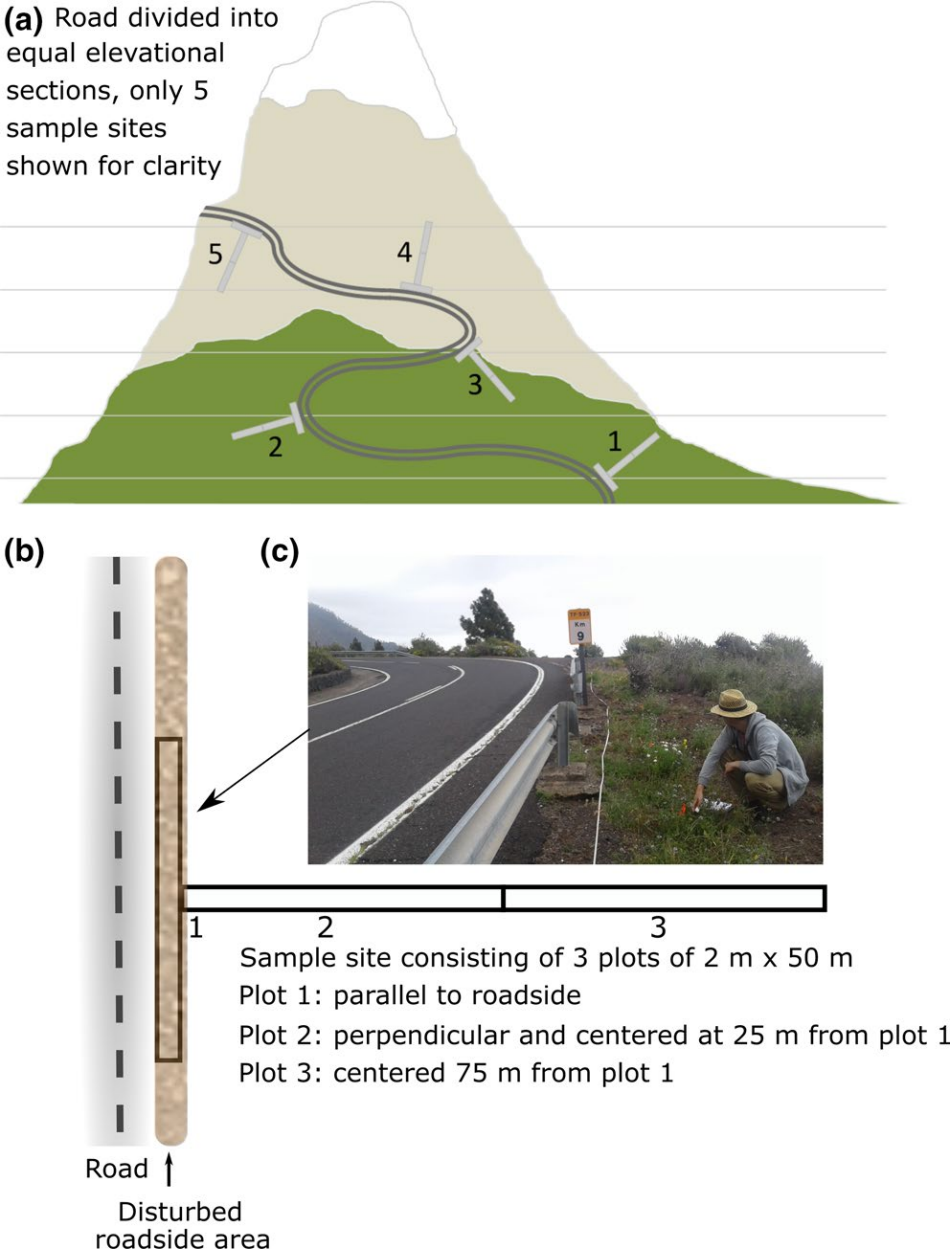
Layout of the MIREN survey design. (a) Equal elevational distribution of 20 sample sites along a mountain road, of which three are selected in each region; (b) Each sample site consists of 3 plots of 2 m × 50 m, plot 1—parallel to the roadside (starting at the first occurrence of roadside vegetation), plot 2—centered 25 m from the roadside plot, plot 3—centered 75 m from the roadside plot; (c) exemplary photograph of monitoring a mountain roadside in Tenerife, Canary Islands, Spain, depicting a survey of plot 1

The MIREN road survey is conducted by region. In each region, the participants select three sample roads that extend over a broad elevation gradient, ideally reaching elevations beyond the treeline (for examples, see Figure 1). We define a region as an area in the same biogeographical unit containing similar flora, geology, and elevational ranges, usually with distances between roads of less than 150 km (Figure 2). Selected roads should begin at the bottom of the mountain region, in a valley, at sea level, or where no further elevation change occurs, and reach the highest elevation typical for roads in the region. Roads can be gravel or paved but should be open for public vehicle traffic for at least some part of the year. Once roads have been selected, the elevational range of each road is divided into 19 equally wide elevational bands from the lowest to highest possible sampling location, giving a total of 20 sample sites per road located at the splits between elevational bands. Sample sites are determined prior to going into the field and located as precisely as possible using a global positioning system (GPS). At each sample site, three 2 m × 50 m plots are laid out in the form of a “T”: one plot (the top of the “T”) is parallel to the road. The other two plots extend end‐to‐end and perpendicular to the road, starting from the center of the first plot, with midpoints at 25 and 75 m from the roadside plot (Figure 3). The same plots are resurveyed every five years. If the plot locations have to be changed due to unforeseen circumstances, new sites are placed as near as possible and again geolocated.

For each plot, a few basic environmental variables have to be collected in the field (e.g., tree cover; see Supporting Information S1). However, we aim to keep the protocol as simple as possible, and therefore additional variables of interest can be either extracted from online resources (e.g., soil characteristics, Hengl et al., 2017) or topographic variables (Amatulli et al., 2018) or through optional add‐on studies (see section below).

### 2.3 Plant species surveys

Within each of the three plots at all 20 sample sites along the three roads, observers record all vascular plant species (including both native and non‐native species) and visually estimate vegetation cover (eight percentage cover classes with 1 = <0.1%, 2 = 0.1–1%, 3 = 2–5%, 4 = 6–10%, 5 = 11–25%, 6 = 26–50%, 7 = 51–75%, 8 = 76–100%) and record abundance (number of individuals in three classes; 1 = 1–10 individuals (or ramets), 2 = 11–100 individuals, 3 = >100 individuals) of each species. The detailed sampling protocol is provided in Supporting Information S1 and can also be downloaded from the MIREN website (www.mountaininvasions.org). Taxa should be identified to species level using up‐to‐date local floras. Before being included in the global database, submitted regional species lists undergo taxonomic harmonization to detect synonyms for the same species in different regions, and to correct spelling problems. This procedure is done by the MIREN data managers, using the R‐packages “taxize” (Chamberlain & Szöcs, 2013; Chamberlain et al., 2020) and “WorldFlora” (Kindt, 2020). First, species names are matched with World Flora Online (http://www.worldfloraonline.org), and if not found there, they are searched via the additional databases included in the Taxonomic Names Resolution Service (Boyle et al., 2013). All changes of species names are transmitted to the submitting region for verification or correction, before the dataset enters the global database (Figure 2).

Each taxon should be classified as native or non‐native to that region by the participant using local floras and databases. As a general rule, plant species introduced into the country or mountain range after AD 1500 are considered as non‐native, although regional deviations are welcome if properly justified. For noteworthy records (e.g., first records or new high/low elevation records of native or non‐native species), specimens should be collected outside of the plots (when possible) and placed in a herbarium to facilitate identification and to inform local floras (Walsh & McDougall, 2018).

### 2.4 Repeated monitoring

To understand long‐term dynamics of redistributions of native and non‐native plant species, all regions should strive for regular long‐term monitoring, preferably with a periodicity of five years (and ideally with at least partial overlap in observers, to reduce observer bias). To facilitate monitoring, all plots should be permanently marked in the field, for instance with magnets or metal tags that can be relocated with a metal detector or colored sticks or plastic seal security tags in remote areas where their removal is unlikely. In addition, precise sub‐meter GPS coordinates should be taken at least once. Photographs should also be taken of each transect to visualize changes over time, document data collection, and facilitate relocation of plots. Surveys and resurveys should always be done at peak biomass or flowering to minimize the risk of missing species with early or late phenology. For repeated surveys, this means that timing should be kept constant relative to the onset of spring, rather than to a fixed date, while sampling within season is recommended from valley bottom to top.

### 2.5 Add‐ons to the standardized protocol

In addition to long‐term monitoring of plant communities, the MIREN survey design is well suited for additional projects (“add‐on” projects) that test more detailed or region‐specific questions about the drivers of plant species redistributions. For example, soil temperatures have been recorded with a high temporal resolution for a year or longer in several MIREN regions to document how disturbance along roadsides affects microclimate, including consequences for species redistributions (for the first regional results, see Lembrechts et al., 2019). Plant functional traits have additionally been collected for species in Tenerife, Canary Islands, to assess contrasting patterns of intraspecific trait variability of native and non‐native species and the change of community mean traits and functional diversity with elevation (Kühn et al., 2021). Another add‐on project has focused on soil chemical properties and mycorrhization of native and non‐native species in the mountains of Norway (Clavel et al., 2021), and survey plots have also been used to assess the distribution of plant pathogens (*Phytophthora* species) in Australia (Khaliq, 2019). Once the participants begin contributing data to the MIREN global road survey database, they can suggest add‐on studies to apply across all regions that go beyond the existing scope of the survey protocol—as long as it is based on a standardized protocol that can be implemented fast, simply, and at low cost by collaborators. To maximize participation and to discuss new proposals, data quality, and complementarity, ideas for add‐on projects should be developed together with the MIREN steering committee.

### 2.6 Data submission and accessibility

The MIREN survey design is a robust and standardized field survey protocol that provides data contributors among others an opportunity to include regional data in research that addresses globally scaled ecological questions. To be included in MIREN’s global road survey database, regional data must be submitted to the MIREN data managers using a standard data format. An overview of how the database is structured and which metadata are stored is provided in Supporting Information S2. While in the first survey in 2007, only non‐native species were monitored, we now only accept data submissions from new regions that surveyed all vascular plant species, both native and non‐native, as partial species pools drastically limit the amount of research questions that can be answered with the data.

All data will be made public in a data repository in the context of paper publications, or at the very latest five years after the survey is undertaken (Figure 2). To date, all survey data collected before 2016 are available through Zenodo (https://doi.org/10.5281/zenodo.5529072). Any researcher can also request the full MIREN database from MIREN data managers for global analyses. The structure of the database, with its plot‐level table with accurate coordinates, and easy linkable species information, allows smooth integration into larger global integrative projects (e.g., data is currently integrated into the SoilTemp‐database; Lembrechts et al., 2020). Details regarding data accessibility and publication, the submission of paper proposals, and guidelines for co‐authorship are given in MIREN’s data‐sharing agreement (see Supporting Information S3), which can also be downloaded from the MIREN website (www.mountaininvasions.org).

## 3 RESULTS

**FIGURE 4:**
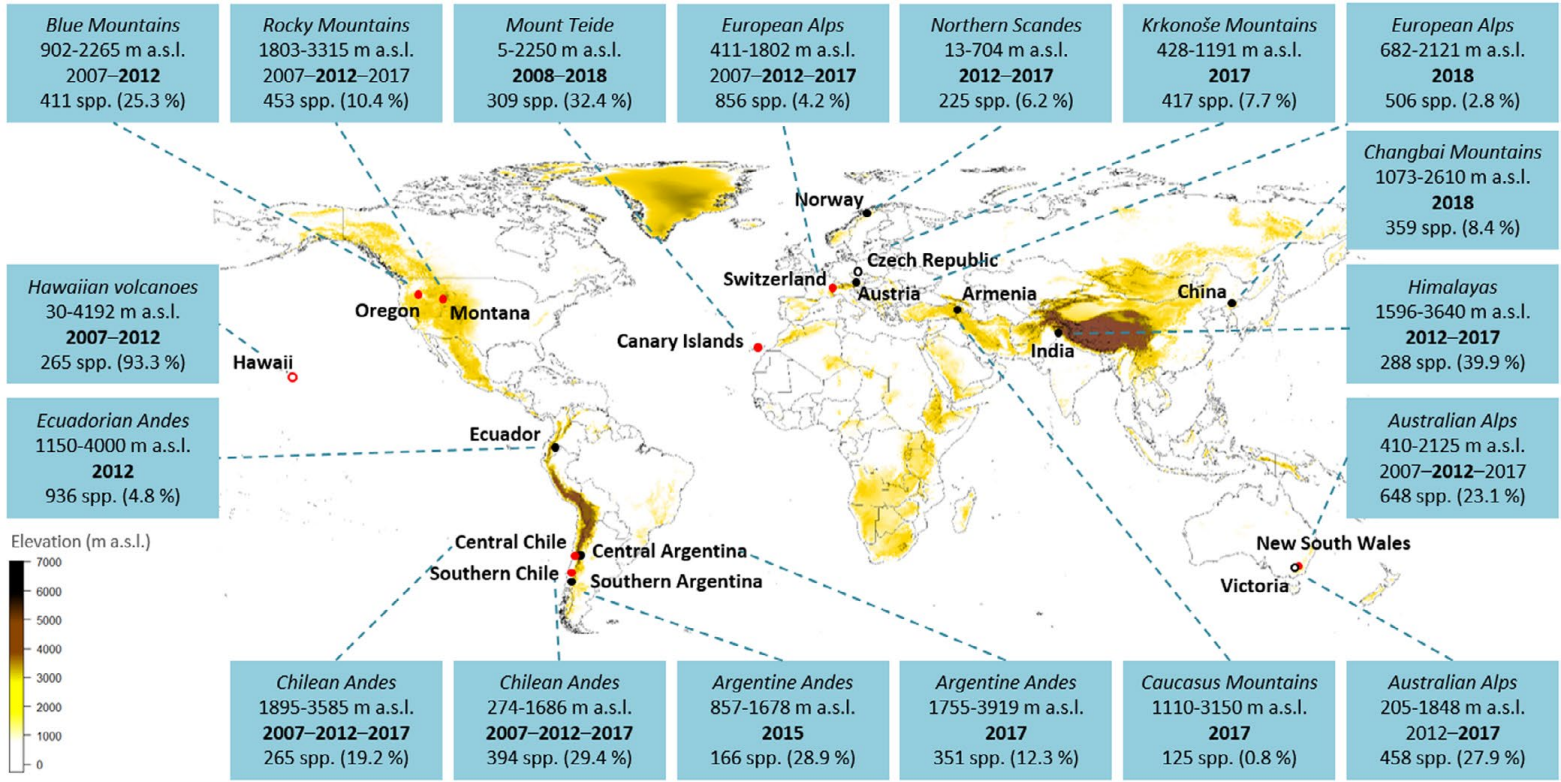
Regions worldwide participating in the vegetation survey along mountain roads according to the standardized protocol of the Mountain Invasion Research Network (MIREN). Red symbols indicate the founding regions from the first survey in 2007. In regions with unfilled symbols, only roadside plots, but not intermediate and interior plots in natural vegetation were sampled. For each region, the name of the mountain range, the sampled elevation gradient and the year(s) of sampling are given. Years in bold indicate that both native and non‐native species were recorded, while in years with normal font only non‐native species were recorded. Note that some regions did not follow the 5‐year sampling frequency. In the last row, the total number of species and in parentheses the proportion of non‐native species are summarized

The standardized protocol for recording plant species communities along mountain roads has been thoroughly tested in the field on all continents except Antarctica (Figure 4). The first survey was carried out in eight regions in 2007 and has been repeated every five years since, resulting in one baseline historical survey (2007) and up to two resurveys (2012 and 2017). The number of regions has increased since 2007, with 18 regions performing the survey by 2018 (Figure 4). The global database currently includes circa 2700 plots and >100,000 observations of >5,000 vascular plant species.

One of the most striking findings of the global MIREN surveys to date has been to document the importance of roads in facilitating mountain invasions. Specifically, we found that non‐native species richness in roadsides decreases with increasing elevation, but generally peaks in the lower third of the elevation gradient (Alexander et al., 2011). In a review including among others the MIREN road survey data, we found that only 2.1% of the non‐native species found in alpine areas can be considered as true alpine or mountain plants based on their temperature affinity (Alexander et al., 2016). Moreover, the vast majority of non‐native species found at high elevation along the MIREN roads are also present at low elevation (Alexander et al., 2011). These findings indicate that non‐native species are first introduced and become established at low elevation sites, from where they spread to higher elevations (Alexander et al., 2011). At higher elevation sites, non‐native species generally become increasingly filtered out by environmental pressures, so few warm‐adapted perennials and mainly generalist species reach higher elevations (McDougall et al., 2018). Recently introduced species may also not have reached their elevational maximum. However, a study from Switzerland demonstrated that non‐native species did not rapidly expand at their high elevation range limits over a period of six years (Seipel et al., 2016). We have also revealed that the number of non‐native species declines with increasing distance from the road (Haider et al., 2018; Seipel et al., 2012), indicating that the native plant community serves as a second environmental filter that selects for more shade‐ and moisture‐tolerant perennials (McDougall et al., 2018). In addition to non‐native species, the MIREN surveys have shown that native species also use roads as corridors (Lembrechts et al., 2017). Interestingly, Lembrechts et al. (2017) found that occurrence optima are located higher in roadside habitats than faraway habitats, and moreover that some alpine species have shifted their ranges downward due to altered abiotic conditions and competitive release in roadside habitats (see also e.g., Lenoir et al., 2010).

Globally, the MIREN surveys have demonstrated that native plant species richness does not follow a consistent pattern in non‐roadside (semi‐) natural habitat along elevation gradients, suggesting the existence of additional region‐specific mechanisms, driven by differences in biome, vegetation type, and human activity. These mechanisms are now the subject of further study. In contrast, a clearer elevation signal is present on roadside plots, with total species richness peaking at mid‐elevations in most regions (Haider et al., 2018). Further, we have observed a reduction in community dissimilarity (beta‐diversity) along roadsides relative to more distant plots, which is amplified by the arrival of non‐native species along mountain roadsides homogenizing plant community composition (Haider et al., 2018). The MIREN surveys have also provided insight into the vulnerability of habitats regionally (Pollnac et al., 2012), the genetic background of successful invasions (Haider et al., 2012) and the impact and management of local invasions (McDougall, Alexander, et al., 2011). For example, in the Greater Yellowstone Ecosystem in the United States, we found that non‐native species emergence varies with elevation and habitat type, which provided land managers valuable information for mitigating biological invasions (Pollnac et al., 2012). Moreover, in the dry Mediterranean Andes in Argentina, which are characterized by treeless vegetation, the survey demonstrated how non‐native plant species can successfully spread from the roadside into natural vegetation at low and intermediate elevations, thus highlighting the susceptibility of these types of ecosystems to invasion (Aschero et al., 2017). By contrast, the alpine vegetation of northern Norway has been shown to be more vulnerable to invasion than its low elevation counterpart, indicating that vegetation structure plays an important role in community invasibility (Lembrechts et al., 2014). Finally, the MIREN surveys have already generated information about regional floras. An excellent example is the discovery of a new species of Poaceae during MIREN monitoring in Kosciuszko National Park, Australia—this species was named after the network: *Poa mireniana* (Walsh & McDougall, 2018).

## 4 DISCUSSION

### 4.1 Strengths of the protocol

**FIGURE 5:**
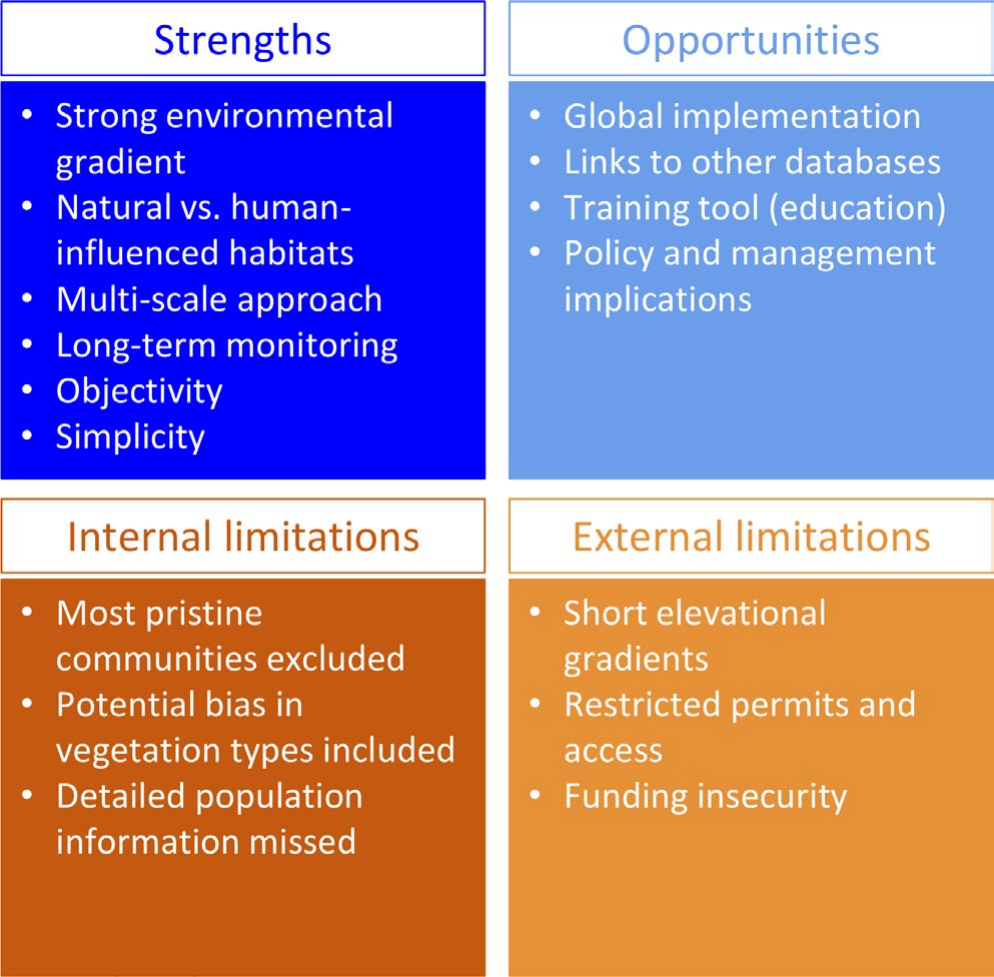
Summary of the strengths and opportunities of the MIREN road survey protocol as well as limitations of the protocol itself and those resulting from external circumstances

The MIREN road survey protocol is unique for its focus on two critical co‐occurring global change drivers of biodiversity and species redistributions in mountains: climate change and road construction (Figure 5). Road construction represents one of the most prominent and increasing land‐use changes in many remote regions (Meijer et al., 2018), leading to physical disturbance, dispersal corridors, and vectors for plant species (Gelbard & Belnap, 2003). Coupled with this, elevation gradients are good proxies for temperature and can be used as space‐for‐time model systems for simulating climate change‐induced temperature increase, where low elevation systems to a certain extent represent future scenarios for higher elevations in a warming climate (Blois et al., 2013; Lembrechts et al., 2017). Given this, combining elevation‐based climate gradients with road effects allows researchers to disentangle the interactive effects of climate and road construction—as an example of human land‐use change—on biodiversity, including their relative importance as drivers of species redistributions. Indeed, it is along clear linear dispersal pathways like roads that changes in species distributions—and especially those of non‐native species—become apparent (Lembrechts et al., 2017). This is particularly relevant when considering the repeated survey approach of the MIREN design, which makes it possible to study the temporal dynamics of plant species distributions in response to natural (e.g., succession after natural disturbances, such as fire), as well as anthropogenic disturbances (e.g., land‐use changes, such as increasing urbanization or domestic grazing, or the introduction of non‐native species), allowing to assess how such disturbances affect the space‐for‐time proxy as would exist along gradients of climatic harshness only.

A final advantage is that along each road, sites are selected at predetermined elevations and capture all habitats found along an elevation gradient, equally covering all elevational belts. The protocol provides a methodological standardization that is straightforward to replicate globally and yet still yields sufficient explanatory power for regional case studies due to its relationship to the elevation gradient and its within‐region replication (i.e., sampling along three mountain roads in each region; e.g., Arévalo et al., 2010; Pollnac et al., 2012; Ratier Backes et al., 2021). In doing so, the protocol remains simple, for example with plots close to roads remaining easy to reach, and thus applicable in many mountain regions even when fieldwork sites need to be easily accessible. This provides another strength of the protocol: it can be repeated in many places, so that general patterns at the global scale can be detected through multi‐region replication (Alexander et al., 2011; Lembrechts et al., 2017; Seipel et al., 2012). In summary, data collected within the MIREN survey framework can be useful for regional and global studies in a large variety of fields, ranging from classical biogeography and community ecology to ecological modeling and global change research.

### 4.2 Limitations of the protocol

The focus of the protocol on mountain roads provides excellent opportunities to disentangle the effects of climate and road construction on plant species and community redistributions. However, the protocol also has four important limitations, which we encourage users to keep in mind when applying the protocol to their study system (Figure 5).

First, the protocol excludes the most pristine environments that exist far from roads and at elevations above where roads reach, so does not monitor mountain biodiversity as a whole. As such, the protocol is a complement to the GLORIA protocol, which focuses on long‐term climate change‐related vegetation shifts on undisturbed mountain summits (Pauli et al., 2015). Nevertheless, one regional study has shown that, at least in northern Scandinavia, the effect of roads on mountain plant diversity could disappear beyond 25 m from the roadside (Lembrechts et al., 2014), and the direct impacts of road disturbance (e.g., construction, maintenance, and vehicle use) are often firmly restricted to the road itself and its shoulders. This suggests that the vegetation monitored in the MIREN survey plot furthest from the roadside (Figure 3b; 50–100 m distance) may indeed at least be free of direct road effects. Yet, using these data beyond the 100 m reach of the sample site could bring issues for some applications, such as spatial modeling, where extrapolations for locations away from the road will suffer from increased uncertainty (Kadmon et al., 2004). Coupled to this, the restriction of the protocol to mountain roads means that, depending on the heterogeneity of the landscape, not all habitat types are necessarily covered relative to their distribution in the ecosystem. Plot locations may be biased toward valleys and less steep terrain if road construction favors such areas. Additionally, while roads represent the most prominent dispersal pathway present in mountains, they are not the only one (e.g., rivers, mountain trails, powerline cuttings, cable cars; Foxcroft et al., 2019). However, the protocol could be easily adapted for other pathways (as done for trails (Liedtke et al., 2020), railroads (Rashid et al., 2021), and rivers (Vorstenbosch et al., 2020)), and we suggest that this would be of particular interest in regions with sparse roads and/or where most of the common non‐native species are wind or water dispersed.

Second, MIREN adopts a discrete temporal and spatial sampling approach. Specifically, since the protocol focuses on community dynamics and large‐scale patterns it has a coarse spatio‐temporal resolution, limited to monitor simple plant community composition estimates over time. The relatively low spatial sampling intensity (i.e., few plots for each elevational belt) and sometimes large distances between elevational increments (on average c. 75 m steps across current MIREN regions, but up to c. 160 m in the Indian Himalayas) can limit understanding of local processes, while also biasing sampling against rarer plant species or habitats. Furthermore, while repeated surveys facilitate investigation of species range dynamics under global change, the complete design does not explicitly consider dispersal dynamics (e.g., through seed rain or seed bank sampling, or seed tracking), instead assessing such dynamics indirectly through repeated snapshots of plant community composition. Additionally, with only one observational moment in a year, the phenological window of observation is small, with the risk of (1) excluding species flowering early or late in the season (Schultz et al., 2014), and (2) confounding phenological shifts over time with distributional shifts (CaraDonna et al., 2014). Whenever possible, we thus encourage regions to survey (a subset of) the plots at three time steps in the season. The latter would also allow for assessing detection probability.

Third, the standard protocol emphasizes simplicity to be as inclusive as possible and to keep resource use to a minimum. The approach thus focuses chiefly on plant community composition and coarse estimates of species abundance (see Supporting Information S1). Other important variables such as biomass, functional traits, community 3D‐structure, species interactions, and other abiotic and biotic variables thus require additional sampling effort. For the same reason, the protocol is limited to vascular plants, excluding bryophytes and other taxonomic groups of potential interest.

Finally, the assumption that elevation can serve as a proxy for climate is of particular relevance here. Testing how the elevation gradient correlates with fine‐grained climatic gradients requires validation using high‐resolution climate data produced either using in‐situ measurements or downscaling of climate models (Lembrechts et al., 2019). We therefore recommend the participants to include at least one add‐on study that deploys temperature data loggers to allow linking of vegetation patterns with microclimatic gradients (Lembrechts et al., 2019)—although this would already add cost.

### 4.3 External limitations

Application of the MIREN road survey protocol might be hindered in some regions, most obviously due to the lack of roads spanning sufficiently large elevation or climatic gradients. Additionally, local land ownership, safety issues, or administrative complexities may complicate establishment and monitoring, for example, on private land or in protected areas (Figure 5). Such issues might be of particular relevance in the MIREN survey design, as MIREN strives to cover a large elevation gradient spanning multiple vegetation zones. At the same time, the proximity of survey plots to roads increases the risk of damage over time (e.g., through road widening, mowing, pesticide use, expanding urbanization, or occasional vandalism). The simplicity of the plot set‐up nevertheless greatly reduces the impact of such damage or vandalism in the long term.

Long‐term monitoring itself comes at a risk of funding insecurities, as the timeframe of 5‐year intervals is beyond what is covered by most grants. Even though maintaining the observational sites themselves comes at virtually no financial cost, the monitoring involves considerable input of field labor, for which costs will vary between regions (Figure 5).

### 4.4 Opportunities

Many drivers of global change act rapidly and interactively, and intensify over time, so assessing their impact on global biodiversity urgently requires comparable data collected on a truly global scale. The MIREN road survey protocol has already demonstrated its potential to explain crucial patterns in native and non‐native species redistributions along mountain roads, but there are a range of further applications that can be explored. For example, due to its simplicity the protocol can readily be implemented in many more mountain ranges and regions. Increasing the number of participating regions, all with their unique combination of climatic conditions and anthropogenic pressures, would further increase the potential to draw general conclusions about the interacting effects of climate change and roads as anthropogenic disturbance on mountain plant communities (Guo et al., 2018). This is particularly important for regions currently under‐represented by the existing MIREN survey sites (Figure 4), such as Africa, Eastern Asia, and central America, regions for which long‐term biodiversity data are often lacking (Maestre & Eisenhauer, 2019). Despite these spatial gaps, MIREN has already more than doubled in size on its road to becoming a global‐scale network since it was first established in eight regions. New participants would thus be able to place their region into a much larger spatio‐temporal picture and, as time passes, get an increasingly strong grasp of how species distributions are changing dynamically, regionally, and across the world.

With its potential to answer important local questions, and feed into the growing multi‐region database, we hope that the MIREN road survey protocol will become the protocol of choice for those interested in native and non‐native plant biodiversity dynamics in mountain regions. At the local scale, it can provide good baseline data on biodiversity changes along elevation gradients in disturbed regions, with opportunities to inform management decisions (McDougall, Alexander, et al., 2011). For example, it can inform policy makers on some of the impacts of urban expansion and new infrastructure projects in mountains, as well as identify new non‐native species before they become problematic. The protocol can also provide essential biodiversity variables for global monitoring efforts (Jetz et al., 2019), since it provides insight into species abundance change over space and time and can further enrich the mountain biodiversity data provided on the online data portal of the Global Mountain Biodiversity Assessment (GMBA). In doing so, it has the capacity to inform global biodiversity policy initiatives, such as the Intergovernmental Science‐Policy Platform on Biodiversity and Ecosystem Services (IPBES).

Further opportunities include add‐ons and expansions to the protocol design, for example to measure microclimate (Lembrechts et al., 2019), dispersal dynamics (e.g., with seed traps), soil biodiversity (e.g., analyses of the soil microbiome or mycorrhizal colonization of roots), or plant‐animal interactions (e.g., pollinator records, herbivore abundance). Collecting such data would be important not only in isolation, but also for helping to create explicit links between descriptive and predictive species distribution models, both at local and global scales. Such efforts could even facilitate modeling of (changes in) the distributions and habitat occupation of mountain plant species, for instance by coupling georeferenced long‐term survey plots with high‐resolution remotely sensed and modeled environmental data (Randin et al., 2020). The survey approach can similarly be expanded by adapting it for use along other linear introduction pathways for non‐native species, such as rivers, railroads or hiking trails (see 4.2 above), or by connecting it with other standardized global biodiversity surveys and assessments, such as GLORIA (Pauli et al., 2015), sPlot (Bruelheide et al., 2019), the Global Inventory of Floras and Traits (GIFT; Weigelt et al., 2020), the Global Naturalized Alien Flora (GloNAF) database (van Kleunen et al., 2015), and the BioTIME database (Dornelas et al., 2018). The connection to other datasets can be done either via the exact geographic location submitted with the MIREN plot‐level data, or via the species names which have been standardized with major taxonomic backbones, such as World Flora Online (http://www.worldfloraonline.org). Finally, the protocol has already shown to have great potential for teaching, for instance by training undergraduate and graduate students in vegetation sampling, while also having relevance for local policy and management, for example as demonstration sites (Figure 5).

## 5 CONCLUSIONS

The MIREN road survey protocol started in 2007 with a specific purpose—to monitor non‐native plant species invasions along mountain roads—but has since then proven to be well‐suited for an increasing number of questions related to species redistributions in the fields of biogeography, ecology, and conservation biology. The protocol is low‐tech, straightforward, and standardized, and can therefore be implemented immediately to fill global gaps in biodiversity data, especially in areas that are traditionally underrepresented in global biodiversity studies (Nuñez et al., 2019) or in regions with scarce or fluctuating government support for scientific research. In short, this on‐the‐ground, multi‐region, simple yet effective monitoring scheme is a perfect example of “Think globally, measure locally,” and has clear capacity to bring together ecologists from around the world to generate an even more complete picture of ongoing species redistributions in mountains. We invite you all to join us!

## CONFLICT OF INTEREST

The authors declare no conflict of interest.

## AUTHOR CONTRIBUTION

**Sylvia Haider:** Conceptualization (lead); Data curation (lead); Methodology (equal); Writing – original draft (lead); Writing – review & editing (lead). **Jonas J. Lembrechts:** Conceptualization (lead); Methodology (equal); Writing – original draft (lead); Writing – review & editing (lead). **Keith McDougall:** Methodology (equal); Writing – original draft (equal); Writing – review & editing (equal). **Aníbal Pauchard:** Methodology (equal); Writing – original draft (equal); Writing – review & editing (equal). **Jake M. Alexander:** Methodology (equal); Writing – original draft (equal); Writing – review & editing (equal). **Agustina Barros:** Methodology (equal); Writing – original draft (equal); Writing – review & editing (equal). **Lohengrin A. Cavieres:** Methodology (equal); Writing – original draft (equal); Writing – review & editing (equal). **Irfan Rashid:** Methodology (equal); Writing – original draft (equal); Writing – review & editing (equal). **Lisa J. Rew:** Methodology (equal); Writing – original draft (equal); Writing – review & editing (equal). **Alla Aleksanyan:** Methodology (equal); Writing – review & editing (equal). **José R. Arévalo:** Methodology (equal); Writing – review & editing (equal). **Valeria Aschero:** Methodology (equal); Writing – review & editing (equal). **Chelsea Chisholm:** Methodology (equal); Writing – review & editing (equal). **Vincent Ralph Clark:** Methodology (equal); Writing – review & editing (equal). **Jan Clavel:** Methodology (equal); Writing – review & editing (equal). **Curtis Daehler:** Methodology (equal); Writing – review & editing (equal). **Pervaiz A. Dar:** Methodology (equal); Writing – review & editing (equal). **Hansjörg Dietz:** Methodology (equal); Writing – review & editing (equal). **Romina D. Dimarco:** Methodology (equal); Writing – review & editing (equal). **Peter Edwards:** Methodology (equal); Writing – review & editing (equal). **Franz Essl:** Methodology (equal); Writing – review & editing (equal). **Eduardo Fuentes‐Lillo:** Methodology (equal); Writing – review & editing (equal). **Antoine Guisan:** Methodology (equal); Writing – review & editing (equal). **Onalenna Gwate:** Methodology (equal); Writing – review & editing (equal). **Anna Hargreaves:** Methodology (equal); Writing – review & editing (equal). **Gabi Jakobs:** Methodology (equal); Writing – review & editing (equal). **Alejandra Jiménez:** Methodology (equal); Writing – review & editing (equal). **Paul Kardol:** Methodology (equal); Writing – review & editing (equal). **Christoph Kueffer:** Methodology (equal); Writing – review & editing (equal). **Christian Larson:** Methodology (equal); Writing – review & editing (equal). **Jonathan Lenoir:** Methodology (equal); Writing – review & editing (equal). **Bernd Lenzner:** Methodology (equal); Writing – review & editing (equal). **Miguel A. Padrón Mederos:** Methodology (equal); Writing – review & editing (equal). **Maritza Mihoc:** Methodology (equal); Writing – review & editing (equal). **Ann Milbau:** Methodology (equal); Writing – review & editing (equal). **John W. Morgan:** Methodology (equal); Writing – review & editing (equal). **Jana Müllerová:** Methodology (equal); Writing – review & editing (equal). **Bridgett Naylor:** Methodology (equal); Writing – review & editing (equal). **Ivan Nijs:** Methodology (equal); Writing – review & editing (equal). **Martin A. Nuñez:** Methodology (equal); Writing – review & editing (equal). **Rüdiger Otto:** Methodology (equal); Writing – review & editing (equal). **Niels Preuk:** Methodology (equal); Writing – review & editing (equal). **Amanda Ratier Backes:** Methodology (equal); Writing – review & editing (equal). **Zafar A. Reshi:** Methodology (equal); Writing – review & editing (equal). **Sabine B. Rumpf:** Methodology (equal); Writing – review & editing (equal). **Verónica Sandoya:** Methodology (equal); Writing – review & editing (equal). **Mellesa Schroder:** Methodology (equal); Writing – review & editing (equal). **Karina Lilian Speziale:** Methodology (equal); Writing – review & editing (equal). **Davnah Urbach:** Methodology (equal); Writing – review & editing (equal). **Graciela Valencia:** Methodology (equal); Writing – review & editing (equal). **Vigdis Vandvik:** Methodology (equal); Writing – review & editing (equal). **Michaela Vítková:** Methodology (equal); Writing – review & editing (equal). **Tom Vorstenbosch:** Methodology (equal); Writing – review & editing (equal). **Tom W. N. Walker:** Methodology (equal); Writing – review & editing (equal). **Neville Walsh:** Methodology (equal); Writing – review & editing (equal). **Genevieve Wright:** Methodology (equal); Writing – review & editing (equal). **Shengwei Zong:** Methodology (equal); Writing – review & editing (equal). **Tim Seipel:** Conceptualization (supporting); Data curation (lead); Methodology (equal); Writing – original draft (equal); Writing – review & editing (lead).

## Supporting information

Supplementary Material S1: MIREN road survey protocol

Supplementary Material S2: Structure of the global MIREN road survey database

Supplementary Material S3: MIREN Data‐sharing agreement

## Data Availability

MIREN road survey data from 2007 to 2015 are available on Zenodo (Seipel, Tim, Haider, Sylvia, & MIREN consortium (2021). MIREN survey of plant species in mountains (v0.1) [Data set]. Zenodo. https://doi.org/10.5281/zenodo.5529072) and through GBIF (https://www.gbif.org/publisher/76388ab6‐61ca‐439a‐ab09‐e1fe73eb224a).
