# Supplementary material for: Think globally, measure locally: The MIREN standardized protocol for monitoring plant species distributions along elevation gradients
Source: Ecol Evol. 2022 Feb 14;12(2):e8590. doi: 10.1002/ece3.8590 (PMC8844121; doi:10.1002/ece3.8590)
Supplement: Supplementary file 1 — Supplementary Material S1: MIREN road survey protocol [file ECE3-12-e8590-s002.pdf]

Road Survey Protocol
of the Mountain Invasion Research Network

Date of this version: 2021-11-21
For more information see www.mountaininvasions.org

1 Overview of the MIREN road survey protocol and contributions

The aim of the Mountain Invasion Research Network (MIREN) road survey protocol is to investigate the changing distribution of native and non-native vascular plant species along elevation gradients in mountain regions around the world. MIREN focuses on the detection of species redistributions due to drivers of global change, such as climate and land-use. Data generated using the standardized protocol described in this document can be used to evaluate and quantify the processes and mechanisms shaping mountain plant communities at regional to global scales. We encourage implementation of the protocol in mountain regions across the globe. For more information about this protocol please also see Haider et al.[1]

2 Protocol methodology

The MIREN road survey uses a stratified approach for recording plant species along mountain roads that traverse the major elevation gradient in a mountainous region (Fig. 1). Stratified sampling occurs within a **Region** along three different **Roads**. Along each Road there are 20 **Sites** evenly stratified by elevation, and at each Site there are three **Plots** at different distances from the road.

2.1 Regions

The delineation of a region will depend on many local factors and we do not seek to constrain these. However, a region must be mountainous as defined by Körner et al. (2017)[2] or the Global Mountain Biodiversity Assessment (GMBA; www.mountainbiodiversity.org). Ideally, a new region should not be in, or part of, an existing region (see www.mountaininvasions.org for an up-to-date map). If you are in doubt about whether your region is suitable for inclusion in the MIREN database (see section 2.6.1), please contact MIREN (miren.contact@gmail.com).

---

[1] Haider S., Lembrechts J.J. et al. (2022). Think globally, measure locally: The MIREN standardized protocol for monitoring plant species distributions along elevation gradients. Ecology & Evolution. https://doi.org/10.1002/ece3.8590.

[2] Körner C. et al. (2017). A global inventory of mountains for bio-geographical applications. Alpine Botany 127, 1–15. https://doi.org/10.1007/s00035-016-0182-6.

2.2 Roads

A road is defined as a corridor which is open to motorized traffic at some point during the year, and which is *not* only used for livestock or as a hiking path. It may be a single corridor or part of a network of corridors. However, each road must continuously connect low and high elevations. Three roads are sampled in each region. Where a choice of roads exists, choose roads which 1) have the largest elevation range, 2) have the most traffic, and 3) capture the geographic and/or environmental variation within the region. Roads should cover a wide elevational range or traverse multiple vegetation zones, ideally including the alpine zone. Defining the bottom of a road is left to the discretion of each group but could be sea level, a place where there is no longer a significant change in elevation, a management boundary (e.g. conservation reserve), or beyond which it is impractical to sample (e.g. urban center). The highest point of the survey should be the highest elevation reached by the road (or accessible by motorized traffic).

2.3 Sites

Sample sites along each road should be evenly stratified by elevation (Fig. 1a). Starting at the bottom, the elevation range of each road is divided into 19 equally spaced bands using digital elevation models or topographic maps, giving 20 sample sites per road. Sample sites are determined *prior* to going into the field to avoid bias, and subsequently located using a global positioning system (GPS). Sites are numbered by elevation along each road from lowest (01) to highest (20). Choose the side of the road to place sample plots at random unless only one side is possible.

When in the field, confirm that the sample site meets the following criteria:

a. The furthest measurements will be required at least 100 m from all roads, so avoid switchbacks, proximity to secondary roads and impassable barriers, e.g. cliff, large river, etc.;

b. It is safe to make measurements at the location (e.g. not on the bend of a busy, narrow road).

2.4 Plots

Each sample site consists of three plots (Fig. 1b), one plot parallel to the roadside (plot 1) and two plots perpendicular to the roadside plot. The intermediate plot (plot 2) adjoins the middle of the roadside plot forming a “T”. The interior plot (plot 3) abuts plot 2 and ends 100 m from the roadside plot (Fig. 1b). Each plot is 2 m x 50 m. The edge of the roadside plot closest to the road should be placed where roadside vegetation begins and sampling can be safely conducted. If time or resources do not permit three plots to be sampled at each site, preference should be given to the roadside plot (plot 1) and the interior plot at 50-100 m from the roadside (plot 3).

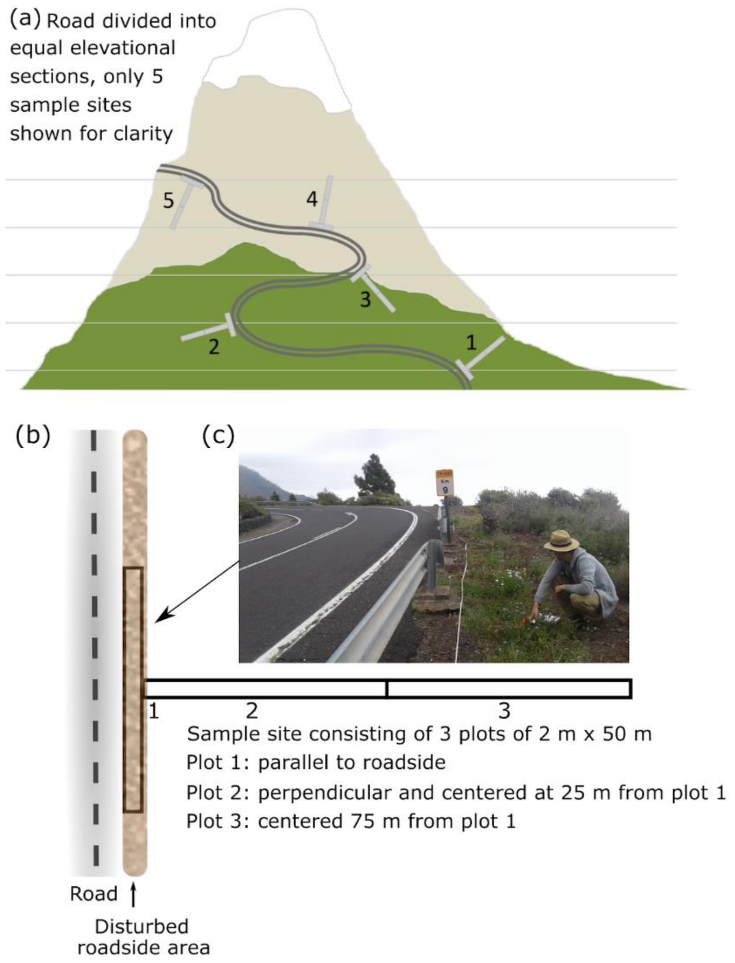

Figure 1. Layout of the MIREN sampling design. (a) Equal elevational distribution of 20 sample sites along a mountain road, of which three are selected in each region; (b) a site consisting of 3 plots of 2 m x 50 m, starting in the disturbed roadside area and extending perpendicularly into the natural vegetation; (c) monitoring of plot 1 in a mountain roadside in Tenerife.

All plots should be permanently marked in the field to facilitate monitoring. Example marking techniques include magnets that can be relocated with a metal detector, colored sticks in remote areas where their removal is unlikely, metal stakes, plastic seal security tags or if physical markers are not possible a combination of hand drawn maps and photos. Very accurate GPSs can also be used to relocate markers. To visualize changes over time and facilitate relocation, photographs should be taken of each plot, ideally with the plot markers visible. This will be especially important for plot 1 (roadside) because the proximity of vegetation to the road may change over time with disturbance. The location of each photo point should be recorded so repeat photos can be taken.

2.5 Data collection

The following descriptions are based on the criteria to include a dataset into the global MIREN database (see section 2.6.1).

All data sheets must include date of survey and names of recorders to facilitate data checking. If you have any questions regarding data collection, contact miren.data@gmail.com.

2.5.1 Site measurements

- *Geolocation* should be recorded at a minimum for each site at the center of the roadside plot (plot 1). It is helpful to record location ends of plots 2 and 3 at 50 and 100 m but this may not be possible if plots 2 and 3 are heavily forested. Geolocation should be reported as latitude and longitude in decimal degrees using the WGS 84 datum; please check that the mapped locations are correct using GIS or Google Earth before submitting data.
- *Elevation:* in meters above sea level; obtained using a digital elevation model or Google Earth; at the same locations as *geolocation* is recorded.

2.5.2 Plot measurements

The following is to be recorded in each 2 m × 50 m plot:

- *Plot code:* is the unique identifier for each plot and is recorded in the form **Region.Road.Site.Plot** (e.g. MTN.BP.06.2). Region should be three letters (e.g. MTN = Montana), and Road a two letter code (e.g. BP = Beartooth Pass Road). Region and Road codes may be altered by the database managers if they have already been used elsewhere. Sites are numbered from 01-20 with 01 being the lowest and 20 the highest. Please include the 0 as a place holder for site codes to ensure character strings of the same length. The roadside plot is numbered 1, the intermediate plot is numbered 2 and the interior plot is 3. If the intermediate plot is not sampled the interior plot should still be recorded as 3.
- *Name and cover of each species:* see section 2.5.2.1 for species nomenclature information and see section 2.5.2.2 for the ordinal projective foliage cover estimates to be used.
- *Abundance of each species* on the following scale: 1 = 1 to 10 individuals (or ramets); 2 = 11 to 100 individuals; 3 = >100 individuals. This coarse scale was used in initial samplings of the global database and its continued use will ensure comparability with previous sampling times.
- *Tree cover:* percent of plot covered by trees taller than 3 m.
- *Bare ground:* percent of plot without vegetation foliage (but excluding rock and litter).
- *Litter:* percent of plot without vegetation foliage but covered with dead and decaying plant material (such as leaves, bark, needles, and twigs)

- *Additional data (optional)*: these can be specific disturbance types, land-use, habitat type, cover of rock, or any other descriptive factors. The data should be provided as text (up to 50 characters) with words separated by an underscore.

2.5.2.1 Species information

All angiosperms, gymnosperms and ferns, both native and non-native, that create foliar cover in a plot are to be recorded. Mosses and lichens can be recorded for regional purposes but are *not* to be submitted to the global database.

If a species can only be identified to family or genus level, a region-specific name should be given. This name is a combination of the taxonomic level, a three-letter region code, and a number (e.g. Acacia MTN1 or Asteraceae MTN2). If species cannot be identified to family level, the name should consist of life form (either graminoid, herb, fern, vine, shrub or tree), the region code and a number (e.g. shrub ORE3).

2.5.2.2 Projective foliage cover estimates

Estimate projective foliage cover of each plant species using the following scale. Projective foliage cover is defined as the proportion of the ground that is shaded by vegetation foliage when lit from directly above. Note that the sum of all species-specific cover estimates can exceed 100 % due to overlap in coverage.

**Cover class**	**1**	**2**	**3**	**4**	**5**	**6**	**7**	**8**
Percentage area	< 0.1	0.1 - 1	2 - 5	6 - 10	11 - 25	26 - 50	51 - 75	76 - 100
Indicative max. area (m x m)	0.1 x 1	1 x 1	1 x 5	1 x 10	1 x 25	1 x 50	1.5 x 50	2 x 50

2.5.3 For repeated observations

For regions repeating the MIREN survey (see also section 2.6.2), relocate the plots used in the previous survey, resample at the same time of year and take new photos. Where it is not possible to resample the same site (e.g. where a major disturbance destroyed the vegetation), establish a new site nearby, and identify the change in the plot code by adding an ‘a’ to the end of the plot code (e.g. MTN.BP.06.2a). When submitting the data inform the data managers that this is a new site. To avoid bias, we suggest sampling without the records from previous years. However, after finishing a plot in the field, the new species records should be compared with the old survey(s) to avoid missing rare species that were recorded previously, and to be consistent in species identification (especially if different people did the previous survey).

2.5.3.1 Survey timing

Sampling should be done at a time that maximizes the chance of recording all species present. This will often be during the peak of flowering or biomass production; i.e. when most species are identifiable. Since flowering at high elevation is often later than at low elevation, it may be expedient to conduct sampling when the highest plots are at their peak of flowering; at that time, low elevation plots will typically be commencing seed production but still identifiable.

2.5.3.2 For repeated observations

For regions repeating the MIREN survey (see also section 2.6.2), relocate the plots used in the previous survey, resample at the optimal time of year for species identification and take new photos. This means that timing should be kept constant relative to the onset of spring, rather than to a fixed date.

Ideally, there is at least partial overlap in observers across survey, to reduce observer bias. Additionally, it is recommended that observers take species lists of previous survey(s) to the field, to assess immediately if species were missed or misidentified. To prevent additional bias emerging from such an approach, we suggest to do a 'blind' sampling, and to compare the new species list only afterwards (but immediately afterwards, to allow repeat recording and corrections).

Whenever possible, regions are encouraged to survey (a subset of) the plots at three time steps in the season to allow for assessing detection probability.

2.6 Data management

Data submitted to the global MIREN database include three spreadsheets:

(1) Species records with the following column headings: a) unique identifier for each plot, b) species name, c) abundance score and d) projective foliage cover class.

(2) A list of all species recorded in each region including family, author and native / non-native status in that region, with data sources for native / non-native status. If the native / non-native status is not known or ambiguous, it should be recorded as 'unknown'.

(3) A metadata-file including all site- and plot-level data listed above.

Templates for these three files can be found on www.mountaininvasions.org or requested from the data managers (miren.data@gmail.com) and *must* be used for submitting data.

A stringent quality control of spelling and synonymy must be undertaken. All species names should be standardized according to the Taxonomic Name Resolution Service (TNRS; http://tnrs.iplantcollaborative.org/) or, if not recorded there, according to a modern, cited flora. Datasets that do not comply with formatting rules will not be incorporated and authors will be notified about which changes are required.

Data should be sent to miren.data@gmail.com.

2.6.1 Inclusion in the MIREN database

Although there is no requirement that data collected using the MIREN road survey protocol is contributed to the MIREN database, we strongly encourage submission of regional datasets. The requirements for data submission are outlined in the MIREN data-sharing agreement document available through www.mountaininvasions.org. Acceptance of data into the database will be at the discretion of the data managers, according to how closely the region has followed the protocol. We strongly advise regions aiming to have their dataset included in the global database, but unable to conduct the complete survey, to contact the data managers *before* starting with the survey. As of January 2021, the MIREN database comprises >100,000 records of >5,000 species in 18 mountain regions around the world.

2.6.2 Observations over time

Some regions have monitored the same plots since 2007 by repeating the survey every five years, enabling evaluation of temporal dynamics. Regardless of when a region does the first survey, to aid data management and preparation of papers using the global dataset we ask that all subsequent surveys be done concurrently: the next repeat survey in the southern hemisphere will be in 2021/22, and in the northern hemisphere in 2022, and then every 5 years.
