# Supplementary material for: Think globally, measure locally: The MIREN standardized protocol for monitoring plant species distributions along elevation gradients
Source: Ecol Evol. 2022 Feb 14;12(2):e8590. doi: 10.1002/ece3.8590 (PMC8844121; doi:10.1002/ece3.8590)
Supplement: Supplementary file 2 — Supplementary Material S2: Structure of the global MIREN road survey database [file ECE3-12-e8590-s003.pdf]

Supporting information S2: Structure of the global MIREN road survey database.

The core database consists of three tables which can be linked to each other. Table A gives information about the cover and abundance of each species in each plot. Through the combination of species name and region, this table can be matched to table B, which provides the status (alien, native, unknown) for each species in the respective region. Table C contains information at the plot or site level, such as geolocation and basic environmental variables like tree cover (indicted here as Envir1 etc.). While some of the information are constant over years (e.g. geolocation), other variables might change with time (e.g. cover of bare soil). Therefore, all three variables of region, plot ID and year have to be used to match tables A and C.

Further species information from add-on studies (e.g. functional traits or mycorrhizal association) can linked easily through table B. Additional plot information (e.g. soil properties or in-situ temperature measurements) can be linked via table C.

This structure has been proven very practical because it does not require from data contributors to be familiar with specific database programs, and for sub-setting and data analyses the tables can simply be handled in the open source statistical environment of R.

(A) Species cover and abundance

Spec.	Region	Plot ID	Year	Cover	Abund.

(B) Species status

Spec.	Family	Region	Status

(C) Environmental data (plot information)

Region	Plot ID	Year	Lat	Long	Elev.	Envir1	Envir2	…

(D) Additionally, we collect metadata per region, which has to be submitted with every (repeated) survey. Metadata is collected primarily as background information and not for analyses. Therefore, they are stored in a separate table D and not included in table C. However, via the variables of region, plot ID and year, metadata can easily be matched to tables A and C. Besides information about data sampling (observer and date), we store information about the regional literature used to determine the species status (see table B) and if plots are located in protected areas (PA).

(D) Metadata (plot scale)

Region	Plot ID	Year	Observ.	Date	Literat.	PA
