# Supplementary material for: Think globally, measure locally: The MIREN standardized protocol for monitoring plant species distributions along elevation gradients
Source: Ecol Evol. 2022 Feb 14;12(2):e8590. doi: 10.1002/ece3.8590 (PMC8844121; doi:10.1002/ece3.8590)
Supplement: Supplementary file 3 — Supplementary Material S3: MIREN Data‐sharing agreement [file ECE3-12-e8590-s001.pdf]

# Data-sharing agreement relating to the use of data collected by the Mountain Invasion Research Network

## Target: Data collected according to the MIREN road survey protocol

Version 1.0
Approved by the MIREN Steering Committee 2020-04-19
For more information see www.mountaininvasions.org

## 1. Data submission

(a) Data submitted to the global MIREN road survey database must be provided in the standardized format created by MIREN. A template is available on www.mountaininvasions.org/?page_id=688 or through an e-mail to miren.data@gmail.com.

(b) Data must be submitted to the MIREN data managers (miren.data@gmail.com).

(c) Data must be cleaned and checked, and will not be accepted if these requirements are not fulfilled.

(d) With submission of their data, the contributing region
- nominates 1 or 2 contact persons recognized as owners of the data, that therefore have the right to submit the data to a global database. These persons will be the “data owners”.
- agrees that the regional data will be harmonized (especially species taxonomy) according to the existing procedure developed by the MIREN data managers based on global taxonomic names.
- agrees that the MIREN Steering Committee has authority to decide whether data from multiple MIREN regions can be used for analyses, based on 1-page proposal to the MIREN Steering Committee.

(e) Submitted data will be used in multi-region analyses.
- The regions give their consent that their data can be used for papers lead by other MIREN data contributors. If a region’s data is used, the regional data owners will be informed by the MIREN Steering Committee. Data contribution will be recognized according to “5. Co-authorship”.
- For accepted paper proposals of non-data contributors (third parties), the data managers will ask the region for data use permission and provide the opportunity to opt out.
- The data managers will never hand over regional data for a single-region paper (data owners can of course use their own data).

(f) Corrections of original datasets must be sent to the MIREN data managers.

(g) Regional datasets may be withdrawn from the global dataset at any time before the data has been published. Already approved paper proposals may still include these data in their analyses. The MIREN Steering Committee decides if a once withdrawn dataset might be re-integrated.

## 2. Data publication

(a) The submitting region agrees that (part of) their data are published as required by journals for paper publications. This can be only species occurrence data with point location (to be published in GBIF; www.gbif.org), or the full dataset used in the analysis (to be published in Dryad; www.datadryad.org).

(b) Time of data publication:
- Species occurrences with point locations (GBIF): earliest publication is 2 years after data were collected. If the first paper is published earlier, the lead author should ask the journal for a moratorium.
- Full dataset used in the analysis (Dryad): In any case, the lead author should ask the journal for a moratorium of 2 years after paper publication to allow time for other papers to be published.

(c) The submitting region agrees that independent of paper publications all species occurrence data with point location will be submitted to GBIF 5 years after data were collected.

## 3. Requests for MIREN road survey data - Paper proposals

(a) The global MIREN road survey dataset might be used by data contributors as well as non-data contributors (third parties). In both cases, a paper proposal needs to be sent to the MIREN co-chairs (miren.chairs@gmail.com; see also www.mountaininvasions.org/?page_id=6).

(b) The paper proposal needs to contain the following (the standardized template created by MIREN must be used):
- Preliminary title
- Brief outline with aims and methods of the study (approx. 0.5 pages)
- A list of authors that will be working with the data, especially those people coming from outside MIREN
- Define a rough timeline
- Specification of required data (which regions, plots, species, years)
- Explicit statement that the applicant agrees with this data-sharing agreement

(c) The MIREN Steering Committee will decide within a month, if the paper proposal is approved, based on the following criteria:
- There is a reasonable link between the aims, expected outputs and data requested.
- There is no conflict with ongoing publication projects using the MIREN database.
- This data-sharing agreement is respected.

(d) The titles of approved paper proposals, together with the names of their applicants, might be published on the MIREN website and in the MIREN newsletter.

(e) If the paper applicant is a data contributor, the data managers will prepare the dataset needed for the planned analyses and in parallel the regions whose data will be used will be informed about the planned paper by the MIREN Steering Committee.

(f) If the paper applicant is not a data contributor, the MIREN Steering Committee will ask all regions whose data were requested, if they are willing to share their data for the specific proposal (opt-out option). Given their agreement (or denial), the data managers will finalize the data set needed for the planned analyses.

(g) After the approval of a paper proposal, the lead author needs to give a short progress report after 6 months. If after 1 year no evidence of progress is given, the applicant needs to ask for proposal renewal, otherwise the proposal is no longer approved, the right to use the data has expired and the topic is open for others.

## 4. Regional analyses and papers

(a) Regional data may of course be used for regional analyses and papers. However, for approved paper proposals, the regions are strongly encouraged to submit regional papers based on the same main idea only after the global paper is accepted by a journal.

## 5. Co-authorship

(a) Opt-out: Each region whose data is used in a paper can nominate one person per survey year as a co-author to acknowledge data contribution (i.e. 1 co-author for the first survey, 2 co-authors for the second survey etc.). Additionally, co-authorship has to be offered to the data managers.

(b) Opt-in: Co-authorship can be earned through substantial intellectual contribution. After approval of a paper proposal and, if needed, approval of data sharing, the MIREN Steering Committee informs the wider MIREN group about the planned analyses. At this point of time, people can state within 4 weeks their interest in becoming a co-author by sending a short statement to the Steering Committee about how they will contribute.

(c) All co-authors should receive at least three opportunities to contribute and review the manuscript. Typically, this should be at the beginning of the project, at the time of the first full manuscript draft, and before paper submission. Opt-in co-authors are expected to contribute at all three stages. Opt-out co-authors are expected to, at least, review and explicitly approve the publication. If a data contributor does not respond to emails approving the final publication, he/she will be removed from the author list (the dataset remains included).

(d) The rules described above are mandatory for all papers based on unpublished data at the time of paper proposal approval. For papers using published data (published full data sets), it is recommended to follow the same criteria.

(e) In any publication making use of MIREN data, the applicants are obliged to give appropriate credit to MIREN. Upon publication, the applicants are requested to send the paper(s) based on MIREN data to the MIREN co-chairs.

## 6. Personal data

(a) According to the General Data Protection Regulation (GDPR; https://gdpr-info.eu) that came into force on 25 May 2018 we herewith give you the opportunity to object to the publication of your name, your public website, or both on the website of the Mountain Invasion Research Network (www.mountaininvasions.org). You can object at the time of data submission, but also at any later point of time. If so, please contact the MIREN coordinators (miren.contact@gmail.com) and your personal information will be removed.
